# Continuing Professional Development ‐ Radiation Therapy

**DOI:** 10.1002/jmrs.673

**Published:** 2023-04-05

Maximise your CPD by reading the following selected article and answer the five questions. Please remember to self‐claim your CPD and retain your supporting evidence. Answers will be available via the QR code and online at www.asmirt.org/news-and-publications/jmrs, as well as published in JMRS – Volume 70, Issue 4 December 2023.

## Radiation Therapy – Original Article

### An evaluation of radiation therapy patient body mass index trends and potential impact on departmental resource planning

Laing B, Caldwell P, Vincent D, Rattray G. (2023) *J Med Radiat Sci*. https://doi.org/10.1002/jmrs.652

1. The World Health Organisation (WHO) defines overweight and obesity as abnormal or excessive fat accumulation that presents a risk to health. According to the WHO, what body mass index (BMI) is considered obese?
  - ≥25 kg/m^2^
  - ≥30 kg/m^2^
  - ≥35 kg/m^2^
  - ≥40 kg/m^2^
2. According to this study, which age category had the highest BMI?
  - 35–44 years of age
  - 55–64 years of age
  - 65–74 years of age
  - 85+ years of age
3. According to this study, which treatment sites showed a statistically significant association with BMI?
  - Brain/skull, abdomen, pelvis/prostate
  - Head and neck, breast, pelvis/prostate
  - Brain/skull, breast, pelvis/prostate
  - Brain/skull, breast, abdomen
4. What does SWL stand for in the article?
  - Safe weight load
  - Safe working limit
  - Safe working load
  - Strong working load
5. Which treatment site demonstrated a statistically significant increase in average BMI, and what was the increase?
  - Breast, 0.31 kg/m^2^ per year
  - Thorax, 0.23 kg/m^2^ per year
  - Thorax, 0.47 kg/m^2^ per year
  - Head and neck, 0.26 kg/m^2^ per year

### Recommended further reading:

1. Seo G, Robinson J, Punch A, Jimenez Y, Lewis S. Understanding radiographic decision‐making when imaging obese patients: A Think‐Aloud study. J Med Radiat Sci. 2022;69(1):13–23.

2. Le NTT, Robinson J, Lewis SJ. Obese patients and radiography literature: what do we know about a big issue? J Med Radiat Sci. 2015;62(2):132–41.

### Answers


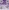


Scan this QR code to find the answers, or visit www.asmirt.org/news-and-publications/jmrs

